# A Panel of Serum MicroRNAs as Specific Biomarkers for Diagnosis of Compound- and Herb-Induced Liver Injury in Rats

**DOI:** 10.1371/journal.pone.0037395

**Published:** 2012-05-18

**Authors:** Yu-Wen Su, Xi Chen, Zhen-Zhou Jiang, Tao Wang, Cheng Wang, Yun Zhang, Jing Wen, Mei Xue, Dan Zhu, Yue Zhang, Yi-Jing Su, Tong-Yue Xing, Chen-Yu Zhang, Lu-Yong Zhang

**Affiliations:** 1 Jiangsu Center of Drug Screening, China Pharmaceutical University, Nanjing, Jiangsu Province, China; 2 State Key Laboratory of Pharmaceutical Biotechnology, School of Life Sciences, Nanjing University, Nanjing, Jiangsu Province, China; 3 Key Laboratory of Drug Quality Control and Pharmacovigilance, China Pharmaceutical University, Ministry of Education, Nanjing, Jiangsu Province, China; 4 Jiangsu Center for Pharmacodynamics Research and Evaluation, China Pharmaceutical University, Nanjing, Jiangsu Province, China; Rutgers University, United States of America

## Abstract

**Background:**

Drug-induced liver injury (DILI) has been a public, economic and pharmaceutical issue for many years. Enormous effort has been made for discovering and developing novel biomarkers for diagnosing and monitoring both clinical and preclinical DILI at an early stage, though progress has been relatively slow. Additionally, herb-induced liver injury is an emerging cause of liver disease because herbal medicines are increasingly being used worldwide. Recently, circulating microRNAs (miRNAs) have shown potential to serve as novel, minimally invasive biomarkers to diagnose and monitor human cancers and other diseases at early stages.

**Methodology/Principal Findings:**

In order to identify candidate miRNAs as diagnostic biomarkers for DILI, miRNA expression profiles of serum and liver tissue from two parallel liver injury Sprague-Dawley rat models induced by a compound (acetaminophen, APAP) or an herb (*Dioscorea bulbifera*, DB) were screened in this study. The initial screens were performed on serum using a MicroRNA TaqMan low-density qPCR array and on liver tissue using a miRCURY LNA hybridization array and were followed by a TaqMan probe-based quantitative reverse transcription-PCR (qRT-PCR) assay to validate comparison with serum biochemical parameters and histopathological examination. Two sets of dysregulated miRNA candidates in serum and liver tissue were selected in the screening phase. After qRT-PCR validation, a panel of compound- and herb- related serum miRNAs was identified.

**Conclusions/Significance:**

We have demonstrated that this panel of serum miRNAs provides potential biomarkers for diagnosis of DILI with high sensitivity and specificity.

## Introduction

Drug-induced liver injury (DILI) is the most frequent reason for the withdrawal of approved drugs from the market and for the rejection of potential drugs in discovery and development phases. DILI directly results in human suffering and health care expenditure [1], [2]. Reports of DILI arouse fear and skepticism in the public regarding the actions of both the pharmaceutical industry and the FDA [3].

Herbal medicines are increasingly used worldwide, and people usually regard them as “dietary supplements” without side effects [4], [5], [6]. With an increasing awareness among healthcare professionals of their potential harmful effects, associated side effects of herbal medicines, including hepatotoxicity, are increasingly reported [4], [5], [6]. In a prospective study from the United States, single or multiple dietary supplements were implicated in 9% of drug-induced liver injury cases [7]. Studies from Asian countries suggest that 20–55% of drug-induced liver injury is related to herbs [8]. As a kind of DILI, there is a growing body of evidence suggesting that herb-induced liver injury is an emerging cause of liver disease [4], [5], [6].

Acetaminophen (APAP), known as paracetamol outside the United States, is the most widely used pharmaceutical analgesic and antipyretic agent across the world and is usually considered to be safe and benign. However, APAP overdose may lead to hepatotoxicity and is potentially fatal [9]. The herb of *Dioscorea bulbifera* (DB), as a traditional Chinese medicine, is widely distributed in Asia and used to treat various diseases, such as thyroid disease and tumors [10], [11]. A series of reports in recent years has demonstrated that DB can cause severe hepatotoxicity in both clinical and preclinical studies [12], [13]. Hepatotoxicity induced either by chemical agents or by herbal medicines has become a public health issue and has limited the clinical applications of such compounds.

Numerous efforts have been made to discover and develop biomarkers that can indicate hepatic disease at early stages. Blood biomarkers in conventional liver function assays, including alanine aminotransferase (ALT) activity, aspartate aminotransferase (AST) activity, alkaline phosphatase (ALP) activity, total bilirubin, gamma-glutamyl transferase (GGT) activity and bile acids, are widely used by physicians to noninvasively evaluate liver injury [3], [14]. However, this set of conventional biomarkers cannot fully meet the needs of clinical diagnosis. For example, serum ALT activity is also associated with muscle necrosis [15], [16]. Serum AST activity is associated with liver toxicity, but also can be elevated in association with heart and skeletal muscle injury [15], [17]. Additionally, serum ALT and AST do not always correlate well with histomorphological results [3]. Although other non-routine predictive biomarkers of hepatotoxicity have received attention recently, they have not been used for routine human clinical testing for various reasons [18].

Recently, the discovery of microRNAs (miRNAs) has paved a new avenue for both the diagnosis and treatment of cancers and other diseases [19], [20]. miRNAs are endogenous non-coding RNAs that are 19–24 nucleotides in length and play an important role in physiological and pathological processes [21]. The expression profiles of highly stable and readily detected circulating miRNAs in serum, plasma, and other body fluids herald immense potential for their use as novel minimally invasive biomarkers in diagnosing and monitoring human cancers [19], [20]. In the process of searching circulating miRNA as diagnostic biomarkers, scientists have developed two working models to identify and refine differentially expressed miRNAs in disease samples compared to control samples [22]: 1) To directly select certain miRNAs as candidates based on the literature data mining. For example, *Laterza et al.* [23] and *Zhang et al.* [24] directly selected certain miRNAs as candidate diagnostic biomarkers for liver injury based on the literature data mining. However, as this approach has no initial high-throughput screening phase, the identified biomarkers might be incomprehensive. 2) To employ some high-throughput techniques (e.g., miRNA microarray, Solexa sequencing and MicroRNA TaqMan low-density arrays) to screen for differentially expressed circulating miRNAs. These high-throughput techniques are widely applied in the initial screening phase and are superior to the existing low-throughput techniques such as northern blotting and cloning [22]. In the validation phase, quantitative real-time PCR (qPCR) methodologies have been widely applied in assessing the low level of certain serum miRNAs in individual samples [25], [26]. For instance, *Wang et al.* [27] employed miRNA microarray to screen for differentially expressed circulating miRNAs in the initial screening phase. However, because microarray and Solexa sequencing are based on sequencing and oligonucleotide hybridization, respectively, the results sometimes do not correlate well with the data of qPCR assay, which is based on the technique of PCR. Thus, the different techniques utilized between initial screening phase and validation phase may result in biased results. To avoid this limitation, some scientists employed an alternative approach which use qPCR based screening techniques (e.g., MicroRNA TaqMan low-density arrays) to identify candidate miRNAs in the initial screening phase [28], [29]. Because the mechanisms of “MicroRNA TaqMan low-density arrays” are same as those of qRT-PCR assay used in the validation phase, the selected candidate miRNAs may be more accurate. Therefore, we employed this approach in present study. Besides, compared with the relative quantification method used in previous studies [23], [24], [27], the absolute quantification strategy we used could enables us to visualize the actual concentrations of circulating miRNAs much more accurately [20], [30].

In the present study, we employed two parallel classic DILI animal models, which were induced by a compound (APAP) and an herb (DB) respectively, and assessed the serum miRNA expression profiles in these two model groups. Our results demonstrate that a new panel of serum miRNAs (miR-122, miR-192 and miR-193) could have the potential to serve as sensitive, specific and noninvasive biomarkers for the diagnosis of DILI.

## Methods

### Animals and animal models

Male 6-week-old Sprague-Dawley rats, which were obtained from the SIPPR-BK Experimental Animal Co., Ltd. (Shanghai, China), were housed in an air-conditioned room with 12/12 h light/dark cycles and were acclimated to the laboratory for 1 week before any experiments were performed with them. Standard rat chow and water were available ad libitum. This study was approved by the Ethical Committee of China Pharmaceutical University, Nanjing University, and Laboratory Animal Management Committee of Jiangsu Province (Approval ID: 2110682). All of the animal experiments were conducted in compliance with the standard ethical guidelines and under the control of the Ethical Committees mentioned above.

APAP (ACROS Organics, New Jersey, USA) in 0.5% carboxymethylcellulose sodium (0.5% CMC-Na) solution was administrated once by oral gavage at 1,600, 800 and 400 mg/kg body weight as described previously [31]. DB water extract in 0.5% CMC-Na solution was administrated once by oral gavage at the doses of 70, 35 and 16 g/kg body weight according to previous description [12] and our validation studies. The vehicle group was administrated with 0.5% CMC-Na solution by oral gavage. For serum miRNA TLDA analysis, serum samples from the vehicle group, 1,600 mg/kg body weight APAP and 70 g/kg body weight DB water extract treatment group were collected at 24 h (n = 10 per group). At the same time point (24 h after administration), serum and liver tissue samples from the vehicle group, control group, 1,600, 800 and 400 mg/kg body weight APAP and 70, 35 and 16 g/kg body weight DB water extract treatment groups were collected for serum miRNA dose-dependent analysis (n = 6 per dose per group) and liver tissue miRNA array analysis (n = 3 per dose per group). Serum samples from the vehicle group, control group, 1,600 mg/kg body weight APAP and 70 g/kg body weight DB water extract treatment group were collected at 3 h, 6 h, 12 h, 24 h, 2 d, 4 d and 7 d after administration for time-dependent analysis (n = 6 per time point per group).

### Blood chemistry

Whole blood samples were centrifuged at 3,000 rpm for 10 min at 4°C about 2 hours after collection at room temperature. Serum samples were transferred into a new tube and stored at −70°C. We used the Olympus AU1000 (Olympus, Japan) automated biochemical analyzer to measure serum biochemical parameters, including ALT and AST, according to the manufacturer's instructions.

### Histopathological examination

Liver biopsy samples for histomorphological evaluation were fixed immediately in 10% neutral-buffered formalin (100 mL 37% aqueous solution of formaldehyde, 4.0 g NaH_2_PO_4_, and 6.5 g Na_2_HPO_4_ per liter), embedded in paraffin, sectioned into 5-µm thick slices, mounted on poly(l-lysine)-treated slides, stained with hematoxylin and eosin, and examined by light microscopy. In our studies conducted to support biomarker qualification, we followed guidelines set by the Society of Toxicologic Pathology on the performance of histopathological evaluations. These guidelines included internal peer review.

### Liver RNA isolation and microarray analysis

Total RNA including miRNA from the vehicle group and 2 DILI model groups (n = 3 per group) were harvested using TRIzol (Invitrogen, Carlsbad, CA, USA) and the RNeasy mini kit (Qiagen, Valencia, CA, USA) according to manufacturer's instructions. After being subjected to RNA measurement on the Nanodrop instrument (the A260/A280 ratio should be close to 2.0 and the A260/A230 ratio should be more than 1.8), 1 µg RNA from each sample was labeled using the miRCURY™ Hy3™/Hy5™ Power labeling kit and hybridized on the miRCURY™ LNA (locked nucleic acid) Array v14.0 (Exiqon, Vedbaek, Denmark). Scanning was performed with the Axon GenePix 4000B microarray scanner. GenePix pro V6.0 was used to read the raw intensity of the image. The intensity of green signal was calculated after background subtraction and the median value of four replicated spots of each probe on the same slide had been calculated. The approach applied for estimating the background of a spot is to select an area near each spot and after identifying the background pixels within this area; the background estimate of the spot is then taken as the sample median of these pixels. We used the median normalization method to obtain “normalized data”; normalized data = (foreground-background)/median with the median being the 50% quantile of microRNA intensity that was greater than 50 in all samples after background correction. All data is MIAME compliant and that the raw data has been deposited in ArrayExpress, Gene Expression Omnibus (Accession Number: E-MEXP-3364).

### Serum miRNA expression profile analysis

The expression levels of miRNA in serum were profiled by stem-loop RT-PCR-based Rodent MicroRNA TaqMan low-density arrays (TLDA) (Applied Biosystems, Foster City, CA, USA). Comprehensive coverage of rodent miRNA species in Sanger miRBase v15 was enabled across a two-card set of TaqMan Array Rodent MicroRNA Cards (Card A v2.0 and Card B v3.0). Serum miRNA collected from 2 DILI groups and the vehicle group (pooling, volume = 10 ml and n = 10 per group) were isolated using the mirVana miRNA Isolation Kit (Applied Biosystems, Foster City, CA, USA). RT-PCR reactions were performed according to the manufacturer's instructions. Briefly, 3 µl total RNA from each sample was reverse transcribed using the TaqMan miRNA Reverse Transcription Kit (Applied Biosystems, Foster City, CA, USA) in combination with Megaplex RT Primers (Rodent Pool Set v3.0) in a total volume of 7.5 µl. The RT cycling conditions were 40 cycles of 16°C for 2 min, 42°C for 1 min and 50°C for 1 sec followed by 85°C for 5 min and a 4°C hold. Pre-amplification was performed using TaqMan PreAmp Master Mix and Megaplex PreAmp Primers (Rodent Pool Set v3.0) (Applied Biosystems, Foster City, CA, USA). Briefly, 2.5 µl of the Megaplex RT products were mixed with 2.5 µl of Megaplex PreAmp Primers and 12.5 µl TaqMan PreAmp Master Mix in a 25-µl PCR reaction. The pre-amplification cycling conditions were 95°C for 10 min, 55°C for 2 min and 72°C for 2 min followed by 12 cycles of 95°C for 15 sec and 60°C for 4 min and followed by 99.9°C for 10 min and a 4°C hold. The pre-amplified cDNA was diluted with 0.1×TE (pH 8.0) to 100 µl, and 9 µl of diluted cDNA was used in each plate for real-time PCR reactions. Quantitative real-time PCR was performed on an Applied BioSystems 7900HT thermocycler using the manufacturer's recommended cycling conditions; cycle threshold (C_T_) values were calculated with the accompanying SDS software v2.3 (Applied Biosystems, Foster City, CA, USA). The relative amount of each miRNA to an internal control was calculated by using the equation 2^−ΔCT^, in which ΔC_T_ = C_T miRNA_−C_T U6_.

### Quantitative Reverse Transcriptional PCR (qRT-PCR) analysis of individual miRNAs

Assays to quantify mature miRNAs in liver tissue were conducted using a Taqman miRNA PCR kit (Applied Biosystems, Foster City, CA, USA) according to the manufacturer's instructions. Briefly, 1 µg of total RNA was reverse-transcribed to cDNA using AMV reverse transcriptase (TaKaRa, Dalian, China) and looped anti-sense primers. The mixture was incubated at 16°C for 15 min, 42°C for 60 min and 85°C for 5 min to generate a library of miRNA cDNAs. Real-time PCR was subsequently performed using an Applied Biosystems 7300 Sequence Detection System (Applied Biosystems, Foster City, CA, USA) and a standardized protocol. In each assay, 1 µL of cDNA was used for amplification. The reactions were incubated in a 96-well optical plate at 95°C for 5 min followed by 40 cycles consisting of a 15 s interval at 95°C and a 1 min interval at 60°C. After the reactions were completed, the C_T_ values were determined using the default threshold settings.

Because U6 RNA and 5S rRNA are degraded in serum samples and a consensus housekeeping miRNA is lacking for qRT-PCR analysis of serum miRNAs, we normalized miRNA concentration to serum volume. Briefly, total RNA was extracted from 100 µL serum with a 1-step phenol/chloroform purification protocol. In brief, 100 µL serum was mixed with 200 µL acid phenol, 200 µL chloroform, and 300 µL diethylpyrocarbonate-treated water. The mixture was vortexed vigorously and incubated at room temperature for 15 min. After phase separation, the aqueous layer was mixed with 1.5 volumes of isopropyl alcohol and 0.1 volumes of 3 M sodium acetate (pH 5.3). This solution was stored at −20°C for 1 h. The RNA pellet was collected by centrifugation at 16,000× g for 20 min at 4°C. The resulting RNA pellet was washed once with 75% ethanol and dried for 10 min at room temperature. Finally, the pellet was dissolved in 20 µL of ribonuclease-free water and stored at −80°C until further analysis. A hydrolysis probe-based qRT-PCR assay was conducted as previously described [25]. We calculated the absolute concentrations of target miRNAs from calibration curves developed with corresponding synthetic miRNA oligonucleotides (Figure S2 and Table S5).

### Statistical analysis

Statistical analysis was performed with SPSS software (version 18). Data are presented as means and standard deviation, two-tailed student's *t*-test was used to determine the statistical differences and *P*-values<0.05 were considered statistically significant. Hierarchical clustering analysis was performed using Multiexperiment Viewer, MeV version 4.7 software (http://www.tm4.org/mev/). The hybridization intensity of each miRNA was median centered, both gene tree and sample tree were clustered, the distance metric was Manhattan Distance, and the linkage method was complete linkage clustering.

## Results

**Figure 1:**
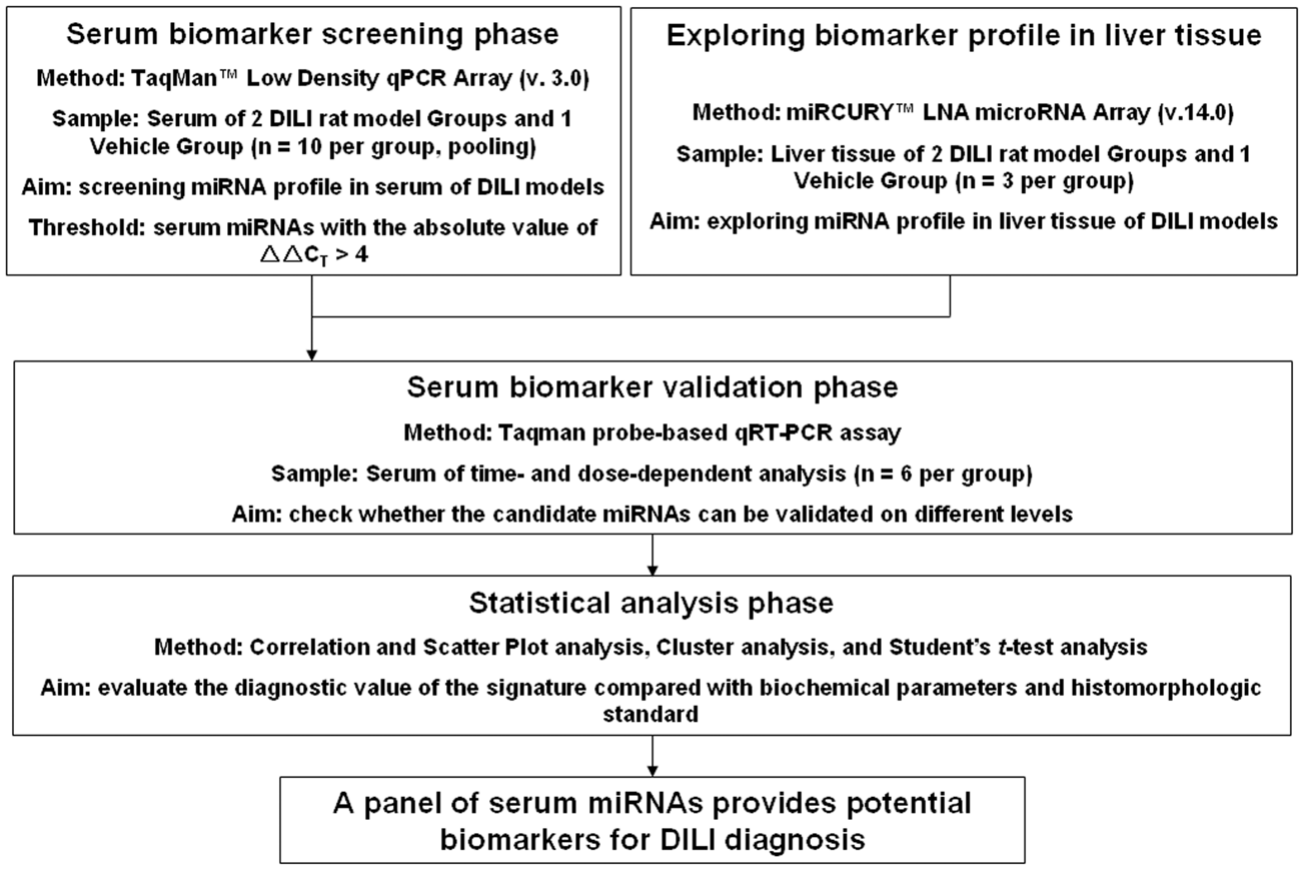
Overview of the experimental strategy.

To obtain DILI-specific miRNA expression profiles of both serum and liver tissue, we employed a strategy using TLDA analysis for serum and hybridization array analysis for liver tissue. Next, we validated the candidate miRNAs as diagnostic biomarkers for DILI on time- and dose-dependent levels in 2 parallel rat DILI models (Figure 1).

### Serum biochemical parameters and histopathological examination results from animal models

Blood chemistry was analyzed in all serum samples collected. Rat serum samples taken 24 h after administration of APAP and DB for miRCURY™ LNA microarray and TLDA analysis showed a significant (*P*-value<0.05) elevation of ALT and AST levels compared with the vehicle serum samples (Table S1). Individually, the ALT and AST levels in each serum sample were 5 times greater than the average limit of normal. This set of serum biochemical parameters correlated strongly with the degree of tissue injury observed through histopathological examination. These results showed that the 2 DILI animal models were successfully established.

### Serum miRNA profiles in rat DILI models explored by TLDA

**Figure 2:**
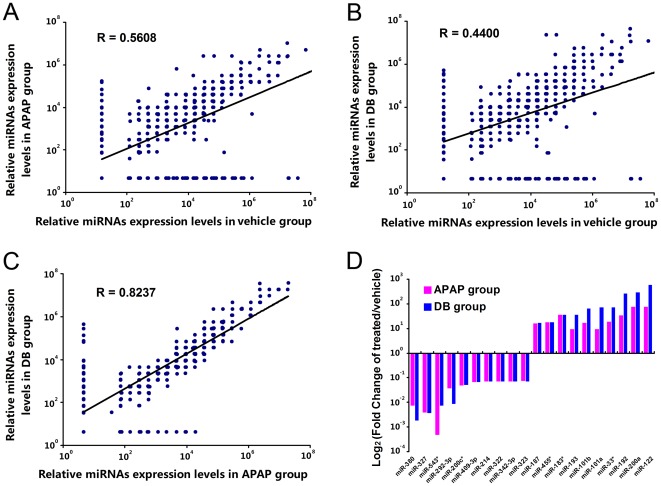
Analysis of serum miRNA expression profiles explored by TLDA. (A) Correlation and scatter plot analysis of relative miRNAs expression levels in APAP group and vehicle group; (B) Correlation and scatter plot analysis of relative miRNAs expression levels in DB group and vehicle group; (C) Correlation and scatter plot analysis of relative miRNAs expression levels in DB group and APAP group; In Panel A, B and C, the relative expression levels of each miRNA to an internal control was calculated by using the equation 2^−ΔCT^×10^7^, in which ΔC_T_ = C_T miRNA_−C_T U6_. The C_T_ value of “Undetectable” was calculated as 40. (D) Top 10 upregulated and downregulated serum miRNAs detected by TaqMan low-density qPCR array. Fold change of treated/vehicle was calculated by using the equation 2^−ΔΔCT^, in which ΔΔC_T_ = (C_T miRNA_−C_T U6_) _DILI group_−(C_T miRNA_−C_T U6_) _vehicle group_. And serum miRNAs were ranked by the fold change of the group with the largest upregulated fold (n = 10 per group, pooling).

The Rodent MicroRNA TaqMan low-density arrays covered all rodent miRNA species in Sanger miRBase v15. The serum miRNA expression profiles revealed by TLDA varied across all three groups. Compared with the vehicle group, 42 serum miRNAs were upregulated and 60 were downregulated (fold change >5 or <0.2) in the 2 DILI model groups (Table S2). Correlation and scatter plot analysis revealed that the correlation coefficients between rat DILI model groups and the vehicle group were low; the R values were 0.5608 and 0.4400 for the APAP-treated group and DB-treated group, respectively (Figure 2A and 2B). As expected, the correlation coefficient between the 2 DILI model groups was much higher with R values as high as 0.8237 (Figure 2C). The top 10 upregulated and downregulated serum miRNAs are highlighted in Figure 2D. These results demonstrated that a set of candidate miRNAs dysregulated in serum could be used as potential diagnostic biomarkers for DILI.

### Liver tissue miRNA profiles in rat DILI models explored by microarray hybridization

miRCURY LNA™ microRNA arrays contain more than 1,700 capture probes, covering all miRNAs annotated in miRBase 14.0 and all viral microRNAs related to rat species. The 2 DILI model groups showed 12 miRNA species that were commonly dysregulated in 2 DILI model groups compared to the vehicle group with a mean fold change >1.3 or <0.77 and a *P*-value<0.05 (Table S3). For the visualization of differentially expressed miRNAs, a heat map was generated using MeV version 4.7 software. The result showed 3 samples from each group clustered in the same tree and a distinct separation of the 2 DILI model groups from the vehicle group. As expected, the 2 DILI model groups clustered in their own tree and clustered together before clustering with the vehicle group (Figure S1). These results suggested that a set of dysregulated liver tissue miRNAs could classify injured liver samples induced by different drugs.

### Validation of dysregulated serum and liver miRNAs by qRT-PCR assay

By individual TaqMan qRT-PCR analysis of dysregulated serum miRNAs uncovered by serum TLDA and dysregulated liver tissue miRNAs uncovered by microarray hybridization in primary screening, 6 serum miRNAs, including miR-122, miR-192, miR-193, miR-200a, miR-21 and miR-29c, exhibited a high correlation with primary screening results. Among this set of serum miRNAs, miR-122, miR-192 and miR-193 presented a significant change in both DILI model groups within the threshold of a fold change >10 and *P*-value<0.05 (Table 1). Besides, validation of the dysregulated liver miRNAs by qRT-PCR showed good correlation with the microarray hybridization results (Table S4). Based on these validation results, a set of extremely and stably dysregulated candidate miRNAs, which could serve as potential diagnostic biomarkers for DILI, were identified.

**Table 1:** qRT-PCR validation of dysregulated serum miRNAs in TLDA results.

miRNAs	APAP group		DB group		
	Fold change	*P*-value	Fold change	*P*-value	
rno-miR-122	54.62	0.00112	255.12	0.00103	significant##
rno-miR-192	16.79	0.00016	24.80	0.00179	significant##
rno-miR-193	50.75	0.00005	46.35	0.00008	significant##
rno-miR-29c	6.03	0.00048	6.77	0.00009	non-significant
rno-miR-21	2.74	0.16797	6.50	0.00055	non-significant
rno-miR-200a	1.86	0.14240	1.83	0.06708	non-significant
rno-miR-183					Assay not linear
rno-miR-101a					Assay not linear
rno-miR-101b					Assay not linear
rno-miR-1188-5p					Assay not linear
rno-miR-133b					Assay not linear
rno-miR-327					Assay not linear
rno-miR-380					undetectable in serum
rno-miR-187					undetectable in serum
rno-miR-206					undetectable in serum
rno-miR-292-3p					undetectable in serum
rno-miR-183*					unchecked
rno-miR-455*					unchecked
rno-miR-33*					unchecked
rno-miR-22*					unchecked
rno-miR-31*					unchecked
rno-miR-200c*					unchecked
rno-miR-543*					unchecked

## : Fold change>10 and *P*-value<0.05 (n = 3).

(The selection of dysregulated serum miRNAs was based on the threshold: the absolute value of ΔΔC_T_>4 in TLDA results. Raw data was presented in Table S2.).

### Time- and dose-dependent analysis of serum miRNAs compared with serum biochemical parameters and histopathological examination results

**Figure 3:**
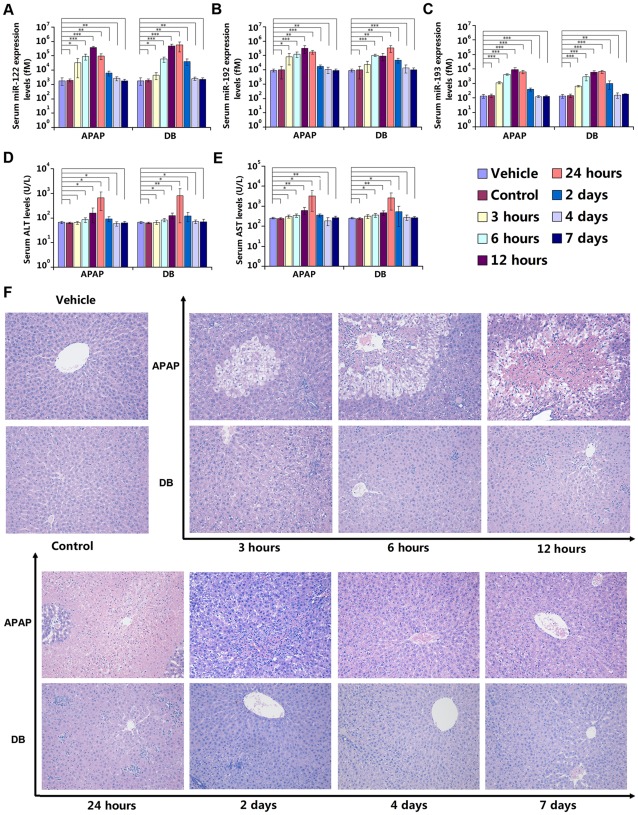
Time-dependent analysis of serum miRNAs compared with biochemical and histopathological parameters. (A) Serum miR-122 expression levels; (B) Serum miR-192 expression levels; (C) Serum miR-193 expression levels; (D) Biochemical parameter: serum ALT levels; (E) Biochemical parameter: serum AST levels. The absolute concentrations of target miRNAs were calculated by referring to calibration curves developed with corresponding synthetic miRNA oligonucleotides. The data were presented as the means ± S.D. and student's *t*-test was used to determine the statistical significance of differences between vehicle group and other groups (*, *P*-values<0.05 *versus* vehicle; * *, *P*-values<0.01 *versus* vehicle; * * *, *P*-values<0.001 *versus* vehicle, n = 6). (F) Histopathological examination results, stained with hematoxylin and eosin, and examined by light microscopy, 200×. Ballooning degeneration appeared in central vein area 3 h after APAP administration, centrilobular necrosis turned out at 6 h and became heavier at 12 h. Hydropic degeneration appeared at 3 h after DB administration, followed by spotty and focal necrosis at 6 and 12 h. The histomorphological changes displayed a recovering manner from 2 d after administration.

In the time-dependent analysis of the serum miRNAs miR-122, miR-192 and miR-193, all of these serum miRNAs exhibited an ascending trend 3 h after administration in both DILI model groups (fold change >2.0); while serum biochemical parameters (e.g., ALT and AST) remained at baseline levels (fold change <1.5). At 6 h after administration, the concentration of these 3 serum miRNAs had risen rapidly (fold change >10.0), while serum biochemical parameters changed slightly (fold change <2.0). The concentration of serum miR-122 peaked at 12 h after administration (fold change >100.0), while serum biochemical parameters showed a limited change (fold change <3.0). Meanwhile, the concentration of the other 2 serum miRNAs changed by a greater degree than serum biochemical parameters (Figure 3A, 3B, 3C, 3D and 3E). The concentration of these 3 serum miRNAs showed a rapid reduction at 2 d after administration, and generally returned to baseline levels from 4 d after administration with a *P*-value>0.05 compared with vehicle group (Figure 3A, 3B and 3C). Serum biochemical parameters displayed a similar fashion to the 3 serum miRNAs (Figure 3D and 3E). In histomorphological examination, ballooning degeneration appeared in central vein area 3 h after APAP administration, centrilobular necrosis turned out at 6 h and became heavier at 12 h. Hydropic degeneration appeared at 3 h after DB administration, followed by spotty and focal necrosis at 6 and 12 h. The histomorphological changes displayed a recovering manner from 2 d after administration (Figure 3F). The changes in both serum miRNAs and biochemical parameters correlated well with the results of histomorphological examination. More importantly, serum miRNA levels are apparently much more sensitive than biochemical parameters are to the administered drugs.

**Figure 4:**
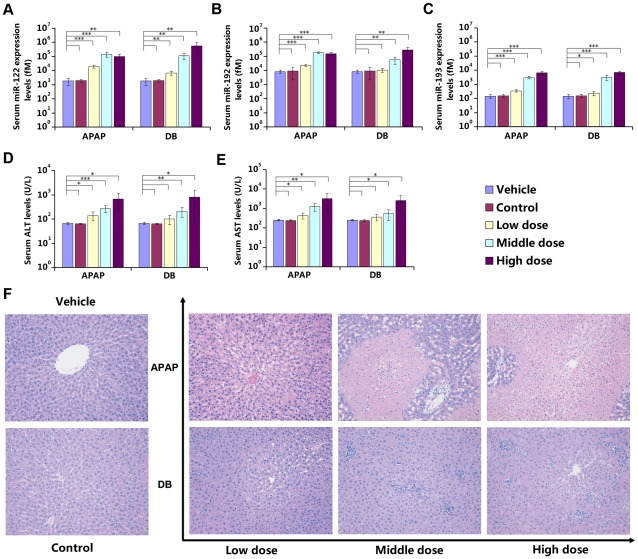
Dose-dependent analysis of serum miRNAs compared with biochemical and histopathological parameters. (A) Serum miR-122 expression levels; (B) Serum miR-192 expression levels; (C) Serum miR-193 expression levels; (D) Biochemical parameter: serum ALT levels; (E) Biochemical parameter: serum AST levels; The absolute concentrations of target miRNAs were calculated by referring to calibration curves developed with corresponding synthetic miRNA oligonucleotides. The data were presented as the means ± S.D. and student's *t*-test was used to determine the statistical significance of differences between vehicle group and other groups (*, *P*-values<0.05 *versus* vehicle; * *, *P*-values<0.01 *versus* vehicle; * * *, *P*-values<0.001 *versus* vehicle, n = 6). (F) Histopathological examination results, stained with hematoxylin and eosin, and examined by light microscopy, 200×. APAP low-dose photomicrograph shown hydropic degeneration, the middle- and high-dose photomicrographs demonstrated submassive and massive necrosis, respectively. DB low-dose photomicrograph shown hydropic degeneration, the middle- and high-dose photomicrographs demonstrated mild and severe focal necrosis, respectively.

In the dose-dependent analysis of the serum miRNAs miR-122, miR-192 and miR-193, miR-122 showed extremely high sensitivity in both 2 DILI model groups (fold change >50.0), while serum biochemical parameters (e.g., ALT and AST) displayed only mild sensitivity (fold change <20.0) in the high-dose group. All 3 serum miRNAs demonstrated better sensitivity than serum biochemical parameters in the middle- and low-dose group, but serum miR-122 was much more sensitive than biochemical parameters (Figure 4A, 4B, 4C, 4D and 4E). In histomorphological examination, APAP low-dose photomicrograph shown hydropic degeneration, the middle- and high-dose photomicrographs demonstrated submassive and massive necrosis, respectively. DB low-dose photomicrograph shown hydropic degeneration, the middle- and high-dose photomicrographs demonstrated mild and severe focal necrosis, respectively (Figure 4F). The changes in both serum miRNAs and biochemical parameters correlated well with the results of histomorphological examination.

The results of these analyses revealed both time- and dose-dependent characteristics of the candidate miRNAs (miR-122, miR-192 and miR-193) as potential diagnostic biomarkers for DILI.

## Discussion

Although the discovery and development of new biomarkers for DILI diagnosis has been regarded as an important issue by the public, the FDA, pharmaceutical industries and research institutions for many years, discovery of sensitive, specific, and noninvasive biomarkers remains at an early stage [3], [18], [32]. Fortunately, since 2008, stable miRNAs have been detected in serum, plasma, urine, saliva and other body fluids, and the expression profiles of these circulating miRNAs have potential as biomarkers for novel and minimally invasive diagnosis and monitoring of human cancers and other diseases including DILI [19], [20], [24], [27]. One of these works carried out by *Wang et al.* [27] directed to the same problem by using different approaches. We have compared both the methodologies and the results between this work and the present one as follows. 1) First of all, we employed two parallel classic DILI animal models, which were induced by a compound (APAP) or an herb (DB), rather than by a single DILI animal model in previous study [27]. The serum miRNA expression profiles of these two model groups were supposed to be more specific to DILI. And the results of correlation and scatter plot analysis supported this assumption well (Figure 2). Beside, among the three serum miRNAs as potential diagnostic biomarker for DILI explored in rat models in present study, miR-122 and miR-192 was also validated in plasma of mice models by *Wang et al.* [27]. This result indicate that circulating miRNAs (whether in serum or in plasma) as potential diagnostic biomarkers for DILI have across-species characteristic as well. In addition, similar results generated via different approaches by these two independent studies makes the conclusion more convincing. 2) In present study, the expression levels of miRNA in serum were profiled by stem-loop RT-PCR-based TLDA, which mechanisms are same as those of qRT-PCR assay used in the validation phase. Therefore, the selection on candidate miRNAs would be more accurate than other initial high-throughput screening techniques, such as solexa sequencing and microarray based on the techniques of sequencing and oligonucleotide hybridization, respectively. While hybridization microarrays were applied in plasma miRNAs profiling by *Wang et al.* [27]. 3) For analyzing individual miRNAs, instead of the relative quantification method used in previous study [27], we applied TLDA and calculated the absolute concentration of target miRNAs by referring to the calibration curves developed with corresponding synthetic miRNA oligonucleotides (Figure S2 and Table S5). 4) In previous study presented by *Wang et al.* [27], the relative expression levels of miRNAs showed an ascending trend at 1 and 3 hours after administration with one single drug. In addition to that, we carried out a time-dependent analysis from 3 hours to 7 days after administration with two drugs independently. The concentration of candidate miRNAs demonstrated not only an ascending trend but also a return to baseline levels following the time (Figure 3). Beyond confirming the early reports, our study demonstrates that a panel of serum miRNAs implicated in compound- and herb-induced liver injury has potential diagnostic value during the development of DILI.

Changes in the serum miRNA profile after drug overdose may reflect a complex tissue-injury pattern [27]. In this study, by employing two parallel rat DILI models, DILI-specific miRNA expression profiles in serum and liver tissue were explored by TLDA (v.3.0) and miRCURY™ LNA array (v.14.0), respectively. The results of correlation and scatter plot analysis supported the assumption that serum miRNA expression profiles of these two model groups were supposed to be more specific to DILI (Figure 2). Among 102 aberrantly expressed serum miRNAs (Fold change >5 or <0.2), 61 displayed the same trend of expression alteration in 2 parallel rat DILI models. Although a significant number of miRNAs in serum were greatly dysregulated, only 12 miRNA species in liver tissue were commonly dysregulated in 2 DILI model groups compared to the vehicle group with a mean fold change >1.3 or <0.77 and a *P*-value<0.05 detected by hybridization. This result was confirmed by TaqMan probe-based qRT-PCR validation. However, the liver tissue miRNA profiles could distinguish the different drug-treated groups clearly in cluster analysis (Figure S1), similar to the application of miRNA profiles in cancer classification [33], [34]. This result suggested that certain liver tissue miRNA profiles could serve as biomarkers for classifying the liver injury induced by different drugs. However, the utility of this type of analysis may be diminished by its invasiveness.

The panel of miRNAs explored in this study all showed decreased expression levels in liver tissue but increased expression levels in serum samples in both parallel rat DILI models (Table 1 and Table S4). ALT activity in the liver is about 3,000 times that of serum activity and the release of ALT from damaged liver cells increases observed ALT activity in the serum [14]. The finding same may be true of miRNAs. The liver-specific miR-122, for example, is over 1,000 times more abundant in 0.1 g liver tissue than in 0.1 mL serum (Figure S3). Although direct leakage of cellular miRNAs into the blood is not common under normal circumstances, it still occurs under conditions of tissue damage or cell apoptosis. Passive leakage from broken cells and tissues is a process that does not require energy. During tumor metastasis or chronic inflammation, tissues or cells at the primary site may break up, thereby releasing miRNAs [22]. Therefore, it is possible to hypothesize that miRNAs in DILI enter the circulation similarly to ALT. Although miRNAs within the panel were abundant in liver tissue (Table S6) and miR-122 was considered liver-specific [24], [35], [36], the liver specificity of other 2 miRNA species in this panel need further analysis.

The panel of aberrantly expressed serum miRNAs (miR-122, miR-192 and miR-193) all exhibited time- and dose-dependent characteristics. Previously, plasma miR-122 and miR-192 had been reported increased linearly from 1 to 3 hours and displayed dose-dependent manner after APAP overdosing in mice. However, by prolonging the observation window up to 7 days after administration with two drugs (APAP and DB) independently in rats, the concentration of 3 serum miRNAs all demonstrated not only an ascending trend but also a return to basal levels following the time and displayed dose-dependent characteristics in two classical DILI rat models. Serum biochemical parameters displayed similar manners to the 3 serum miRNAs and the changes in both serum miRNAs and biochemical parameters correlated well with the features of histomorphological examination (Figure 3 and Figure 4). Owing to the preanalytical steps which could affect miRNA detection and quantification, only miRNAs that are extremely up- or downregulated would be suitable as clinical biomarkers [37]. The 3 miRNAs within the panel were all extremely upregulated and, therefore, suitable as potential clinical DILI diagnosis biomarkers. Given that circulating miR-122 was previously reported as a DILI biomarker, the results from the strategies in this study indicate that there is a set of serum miRNAs highly related to DILI. Among them, circulating miR-122 had been reported as liver-specifically conserved across species [24], [35], [36] and has been applied to diagnosis of various liver diseases in clinical studies, including hepatocellular carcinoma (HCC) [38], [39], [40], hepatitis B virus (HBV) infection [24], [39], cirrhosis [38] and alcohol- and chemical-related hepatic diseases [24], [27]. Besides, unlike ALT and AST, miR-122 has no association with muscle disorders [24]. Unfortunately, this approach has its own disadvantages; circulating miR-122 could not serve as a biomarker to distinguish these different liver diseases. Once an elevated circulating miR-122 level has been detected, a physician cannot make a judgment about what the specific liver disease(s) is/are using this information alone. Patients with circulating miR-122 elevation might be infected by HBV and/or present HCC and/or be stimulated by alcohol abuse rather than drug overdosing. However, employing a miRNA expression panel instead of an individual miRNA as a biomarker represents a rational option to circumvent the current limitations in using miRNAs for DILI diagnosis [41]. If DILI-specific miRNAs within the panel were all aberrantly expressed in the same time, a physician could more easily and accurately judge whether patients have DILI. In order to identify DILI-specific serum miRNAs released from drug-injured liver tissue rather than from other organs or other hepatic injuries, such as viral-induced hepatic injury or hepatic cancer, we intend to perform large-scale tests of hepatic toxicants and different species of model animals to solve this problem. Moreover, in the validation phase, the candidate miRNAs as potential biomarkers for DILI diagnosis were selected from the miRNAs that were dysregulated in both 2 DILI groups (listed in bold characters in Table S2). Since the selected miRNAs are common in both groups, they can not discriminate APAP group from DB group. Future studies may be necessary to directly compare the differentially expressed miRNAs between APAP group and DB group.

Contemporary hepatotoxicity biomarker discovery efforts focused on marker proteins or metabolites from animal or human body fluids, from animal tissue extracts/supernatants or from primary hepatocyte cell cultures have been limited in clinical use because of their low-throughput nature [18]. Although progress in proteomics and metabolomics has led to the development and high-throughput use of mass spectrometry (MS) and nuclear magnetic resonance (NMR), technologies that have the potential to identify unknown proteins in plasma and urine samples, such assays are expensive and labor-intensive, which hinders their widespread application in clinical diagnosis [18], [32], [41]. The enzyme-linked immunosorbent assay (ELISA) can be performed in a high-throughput manner and is suitable for the detection of diagnostic antigens, but its detection sensitivity is greatly dependent on antibody affinities [18], [32], [41]. As circulating miRNAs may be ideal biomarker candidates for various cancers and other diseases, great attention has been devoted to the discovery of liver disease-related miRNA biomarkers [27]. Advantages of miRNAs as biomarkers include noninvasiveness, stability in body fluids, lower complexity, no post-processing modification, a tissue-restricted expression profile, synthetic high-affinity “capture” reagent and “amplifiable” signals [20], [27], [41].

In summary, serum miR-122, miR-192 and miR-193 constitute a new panel for compound- and herb-induced liver injury diagnosis. To conclude that this panel of miRNAs is a diagnostic biomarker for DILI, cross-species validation, screens of additional hepatotoxicants and clinical trials need to be conducted in the future. Nevertheless, the panel of miRNAs uncovered in the present study provides potential biomarkers for diagnosis of DILI with high sensitivity and specificity.

## Supporting Information

Figure S1 Hierarchical clustering analysis of commonly dysregulated liver miRNAs explored by microarray hybridization. 12 liver miRNA species that were commonly dysregulated in 2 DILI model groups compared to the vehicle group with a mean fold change >1.3 or <0.77 and a *P*-value<0.05 were applied to clustering analysis. The hybridization intensity of each miRNA was median centered, both gene tree and sample tree were clustered, the distance metric was Manhattan Distance, and the linkage method was complete linkage clustering. Rows represented miRNA species, and columns represented the samples from vehicle group and 2 DILI model groups. Color areas indicated relative expression levels of each miRNA compared with median expression level (red, above the median level; green, below the median level; and black, close to the median level). The dendrogram displayed a clear separation of not only 2 DILI liver samples from the vehicle liver samples but also APAP-treated liver samples from DB treated liver samples (n = 3).
(TIF)

Figure S2 Calibration curves of serum miRNA qRT-PCR analysis. (A) Calibration curve of miR-122; (B) Calibration curve of miR-192; (C) Calibration curve of miR-193. Synthetic miRNAs oligonucleotides of known concentrations were reverse-transcribed and amplified (n = 3).
(TIF)

Figure S3 miRNA expression levels in serum and liver tissue of control group. (A) miR-122 expression levels; (B) miR-192 expression levels; (C) miR-193 expression levels. The volume of liver was converted by the equation: volume (L) = weight (mg)÷density (mg/dL)÷10 (dL/L). Ratio: miRNA concentration in liver versus in serum (n = 3).
(TIF)

Table S1 Serum biochemical parameters of rat model serum samples.
(DOC)

Table S2 Dysregulated serum miRNAs in 2 DILI groups comparing with vehicle group.
(DOC)

Table S3 Dysregulated liver tissue miRNAs in 2 DILI groups compared with vehicle group.
(DOC)

Table S4 qRT-PCR validation of dysregulated liver miRNAs in microarray hybridization results.
(DOC)

Table S5 Sequences of synthetic mature miRNAs.
(DOC)

Table S6 Top 20 abundant miRNA species of liver tissue in microarray hybridization results.
(DOC)
