# Supplementary figures and images for: A Panel of Serum MicroRNAs as Specific Biomarkers for Diagnosis of Compound- and Herb-Induced Liver Injury in Rats
Source: PLoS One. 2012 May 18;7(5):e37395. doi: 10.1371/journal.pone.0037395 (PMC3356255; doi:10.1371/journal.pone.0037395)

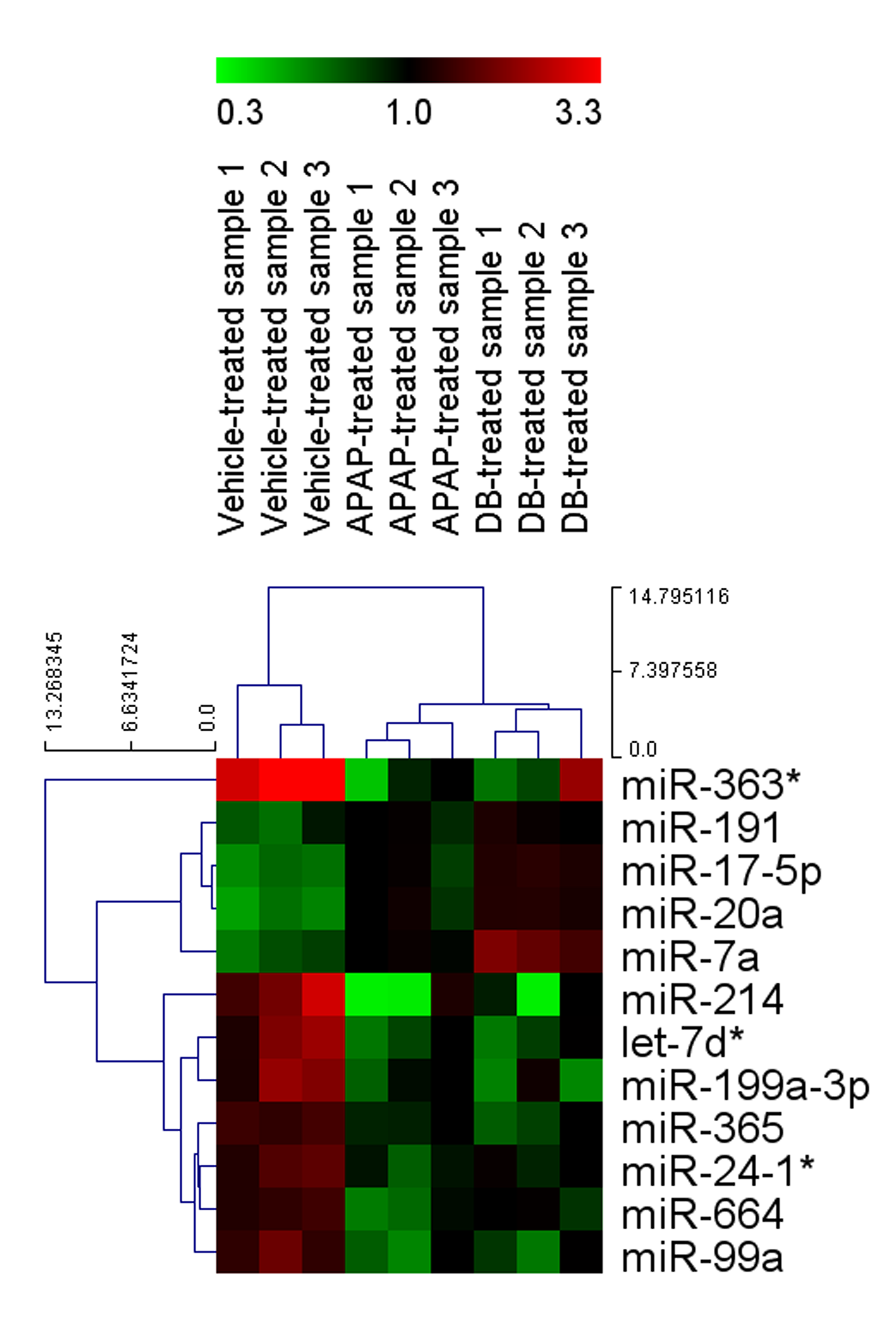

Supplement: Figure S1 — Hierarchical clustering analysis of commonly dysregulated liver miRNAs explored by microarray hybridization. 12 liver miRNA species that were commonly dysregulated in 2 DILI model groups compared to the vehicle group with a mean fold change >1.3 or <0.77 and a P-value<0.05 were applied to clustering analysis. The hybridization intensity of each miRNA was median centered, both gene tree and sample tree were clustered, the distance metric was Manhattan Distance, and the linkage method was complete linkage clustering. Rows represented miRNA species, and columns represented the samples from vehicle group and 2 DILI model groups. Color areas indicated relative expression levels of each miRNA compared with median expression level (red, above the median level; green, below the median level; and black, close to the median level). The dendrogram displayed a clear separation of not only 2 DILI liver samples from the vehicle liver samples but also APAP-treated liver samples from DB treated liver samples (n = 3). (TIF) [file pone.0037395.s001.tif]

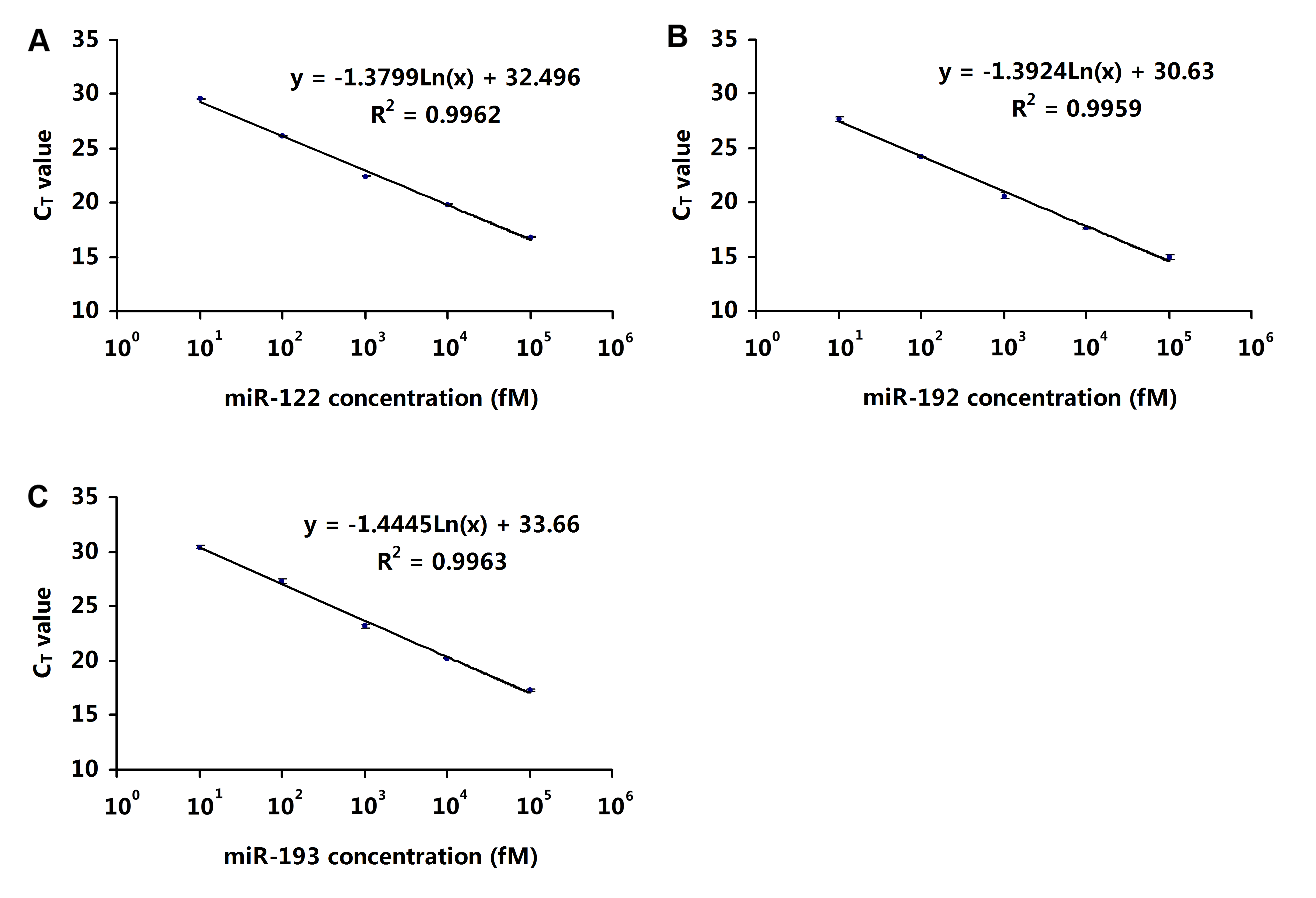

Supplement: Figure S2 — Calibration curves of serum miRNA qRT-PCR analysis. (A) Calibration curve of miR-122; (B) Calibration curve of miR-192; (C) Calibration curve of miR-193. Synthetic miRNAs oligonucleotides of known concentrations were reverse-transcribed and amplified (n = 3). (TIF) [file pone.0037395.s002.tif]

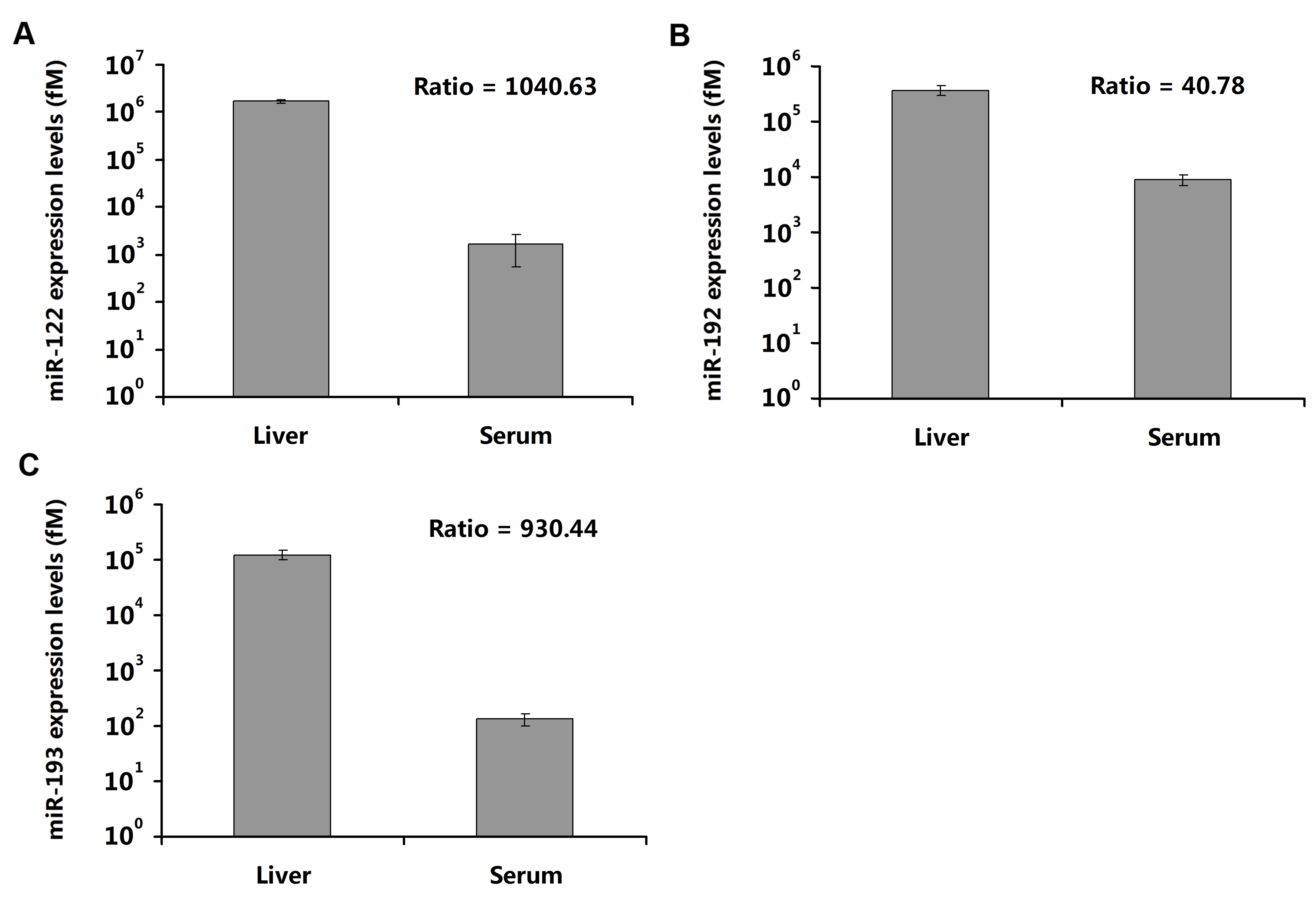

Supplement: Figure S3 — miRNA expression levels in serum and liver tissue of control group. (A) miR-122 expression levels; (B) miR-192 expression levels; (C) miR-193 expression levels. The volume of liver was converted by the equation: volume (L) = weight (mg)÷density (mg/dL)÷10 (dL/L). Ratio: miRNA concentration in liver versus in serum (n = 3). (TIF) [file pone.0037395.s003.tif]
