# Supplementary material for: A Panel of Serum MicroRNAs as Specific Biomarkers for Diagnosis of Compound- and Herb-Induced Liver Injury in Rats
Source: PLoS One. 2012 May 18;7(5):e37395. doi: 10.1371/journal.pone.0037395 (PMC3356255; doi:10.1371/journal.pone.0037395)
Supplement: Table S1 — Serum biochemical parameters of rat model serum samples. (DOC) [file pone.0037395.s004.doc]

**Supplementary Data Table 1.** Serum biochemical parameters of rat model serum samples (Mean ± SD, n = 13)

|  | **ALT (U/L)** | **AST (U/L)** |
| --- | --- | --- |
| Vehicle | 78.5±4.5 | 284.7±40.8 |
| APAP | 780.4±662.5  (8.30×10^-4*^) | 4613.9±3960.6  (6.12×10^-4*^) |
| DB | 1092.7±661.2  (1.09×10^-5#^) | 5999.7±4251.9  (6.14×10^-5#^) |

*: *P*-value, APAP group vs. Vehicle group; #: *P*-value, DB group vs. Vehicle group.
