# Supplementary material for: A Panel of Serum MicroRNAs as Specific Biomarkers for Diagnosis of Compound- and Herb-Induced Liver Injury in Rats
Source: PLoS One. 2012 May 18;7(5):e37395. doi: 10.1371/journal.pone.0037395 (PMC3356255; doi:10.1371/journal.pone.0037395)
Supplement: Table S2 — Dysregulated serum miRNAs in 2 DILI groups comparing with vehicle group. (DOC) [file pone.0037395.s005.doc]

**Supplementary Data Table 2.** Dysregulated serum miRNAs in 2 DILI groups comparing with vehicle group (Fold Change >5 or < 0.2).

| **Upregulated**  **miRNAs** |  | **CT** |  |  | **△Ct** |  | **Fold change** | |
| --- | --- | --- | --- | --- | --- | --- | --- | --- |
|  | **Vehicle** | **APAP** | **DB** | **Vehicle** | **APAP** | **DB** | **APAP** | **DB** |
| **rno-miR-122** | 28.99 | 20.97 | 17.98 | 8.29 | 2.08 | -0.88 | 74.029 | 575.427 |
| **rno-miR-200a** | 35.99 | 27.97 | 25.97 | 15.29 | 9.08 | 7.11 | 74.443 | 290.619 |
| **rno-miR-192** | 27.85 | 20.99 | 17.96 | 7.15 | 2.09 | -0.90 | 33.294 | 264.984 |
| **rno-miR-33*** | 36.00 | 29.99 | 27.97 | 15.30 | 11.10 | 9.11 | 18.459 | 73.146 |
| **rno-miR-101a** | 31.96 | 26.94 | 23.97 | 11.26 | 8.05 | 5.11 | 9.274 | 71.147 |
| **rno-miR-101b** | 32.88 | 26.98 | 25.00 | 12.18 | 8.08 | 6.14 | 17.085 | 65.479 |
| **rno-miR-193** | 35.96 | 30.93 | 28.94 | 15.27 | 12.03 | 10.08 | 9.404 | 36.315 |
| **rno-miR-183*** | 36.97 | 29.99 | 29.95 | 16.27 | 11.09 | 11.09 | 36.097 | 36.283 |
| **rno-miR-22*** | 33.94 | 28.98 | 26.97 | 13.24 | 10.09 | 8.11 | 8.890 | 35.013 |
| **rno-miR-455*** | 35.03 | 29.03 | 29.04 | 14.33 | 10.14 | 10.18 | 18.239 | 17.742 |
| **rno-miR-183** | 35.98 | 30.90 | 29.99 | 15.28 | 12.01 | 11.13 | 9.644 | 17.646 |
| **rno-miR-31*** | 32.96 | 29.95 | 27.02 | 12.26 | 11.06 | 8.16 | 2.305 | 17.212 |
| **rno-miR-187** | 36.89 | 31.10 | 31.01 | 16.19 | 12.21 | 12.15 | 15.803 | 16.480 |
| **rno-miR-878** | 34.96 | 30.00 | 29.93 | 14.26 | 11.11 | 11.07 | 8.871 | 9.139 |
| **rno-miR-1188-5p** | 32.88 | 27.06 | 27.87 | 12.18 | 8.16 | 9.01 | 16.180 | 8.991 |
| **rno-miR-194** | Undet | 26.16 | 23.98 |  |  |  |  |  |
| **rno-miR-374** | Undet | 29.91 | 24.03 |  |  |  |  |  |
| **rno-miR-802** | Undet | 29.98 | 26.98 |  |  |  |  |  |
| **rno-miR-632** | Undet | 28.95 | 29.99 |  |  |  |  |  |
| **rno-miR-381** | Undet | 29.96 | 29.99 |  |  |  |  |  |
| **rno-miR-362*** | Undet | 33.95 | 29.95 |  |  |  |  |  |
| rno-miR-29c | 33.98 | 29.99 | 25.98 | 13.28 | 11.10 | 7.12 | 4.531 | 71.615 |
| rno-miR-21 | 27.99 | 25.98 | 20.97 | 7.29 | 7.09 | 2.11 | 1.151 | 36.325 |
| rno-miR-500 | 33.05 | 28.99 | 27.99 | 12.35 | 10.10 | 9.13 | 4.766 | 9.305 |
| rno-miR-182 | 33.99 | 30.00 | 28.96 | 13.29 | 11.11 | 10.11 | 4.532 | 9.085 |
| rno-miR-195 | 29.02 | 27.00 | 23.99 | 8.32 | 8.10 | 5.14 | 1.160 | 9.074 |
| rno-miR-152 | 27.98 | 24.96 | 22.96 | 7.28 | 6.06 | 4.10 | 2.320 | 9.049 |
| rno-miR-29c* | 34.99 | 31.01 | 29.98 | 14.30 | 12.12 | 11.12 | 4.526 | 9.024 |
| rno-miR-31 | 29.97 | 27.98 | 24.97 | 9.27 | 9.08 | 6.12 | 1.141 | 8.916 |
| rno-miR-27b | 29.96 | 26.96 | 24.97 | 9.26 | 8.06 | 6.11 | 2.302 | 8.878 |
| rno-miR-26b | 28.96 | 26.98 | 23.98 | 8.26 | 8.09 | 5.12 | 1.129 | 8.814 |
| rno-miR-151 | 35.92 | 33.05 | 30.96 | 15.22 | 14.16 | 12.10 | 2.087 | 8.688 |
| rno-miR-22 | 25.99 | 24.98 | 21.03 | 5.29 | 6.08 | 2.17 | 0.576 | 8.681 |
| rno-miR-200b | 29.96 | 26.98 | 25.01 | 9.26 | 8.08 | 6.15 | 2.255 | 8.625 |
| rno-miR-505 | 32.87 | 28.96 | 27.96 | 12.17 | 10.07 | 9.10 | 4.293 | 8.397 |
| rno-miR-370 | 33.91 | 30.04 | 29.00 | 13.21 | 11.15 | 10.14 | 4.177 | 8.365 |
| rno-miR-193* | 30.89 | 26.97 | 26.00 | 10.19 | 8.08 | 7.14 | 4.335 | 8.311 |
| rno-miR-532-5p | Undet | Undet | 25.93 |  |  |  |  |  |
| rno-miR-503 | Undet | Undet | 28.95 |  |  |  |  |  |
| rno-miR-743a | Undet | Undet | 28.98 |  |  |  |  |  |
| rno-miR-431 | Undet | Undet | 29.02 |  |  |  |  |  |
| rno-miR-490 | Undet | Undet | 29.91 |  |  |  |  |  |
| **Downregulated**  **miRNAs** |  | **CT** |  |  | **△CT** |  | **Fold change** | |
|  | **Vehicle** | **APAP** | **DB** | **Vehicle** | **APAP** | **DB** | **APAP** | **DB** |
| **rno-miR-380** | 28.70 | 33.99 | 35.98 | 8.00 | 15.09 | 17.12 | 0.007 | 0.002 |
| **rno-miR-327** | 23.76 | 30.00 | 29.99 | 3.06 | 11.10 | 11.13 | 0.004 | 0.004 |
| **rno-miR-543*** | 26.76 | 35.99 | 32.01 | 6.06 | 17.09 | 13.15 | 0.001 | 0.007 |
| **rno-miR-292-3p** | 30.01 | 32.97 | 35.00 | 9.31 | 14.07 | 16.14 | 0.037 | 0.009 |
| **rno-miR-200c*** | 28.47 | 31.00 | 30.97 | 7.77 | 12.11 | 12.11 | 0.049 | 0.050 |
| **rno-miR-484** | 23.90 | 24.96 | 25.97 | 3.20 | 6.07 | 7.11 | 0.137 | 0.066 |
| **rno-miR-409-3P** | 29.96 | 30.99 | 31.98 | 9.26 | 12.10 | 13.12 | 0.140 | 0.069 |
| **rno-miR-128a** | 25.98 | 26.97 | 27.98 | 5.28 | 8.08 | 9.12 | 0.144 | 0.070 |
| **rno-miR-322** | 27.00 | 29.02 | 28.98 | 6.30 | 10.12 | 10.13 | 0.071 | 0.071 |
| **rno-miR-342-3p** | 22.00 | 23.99 | 23.96 | 1.30 | 5.09 | 5.10 | 0.072 | 0.072 |
| **rno-miR-323** | 30.99 | 32.98 | 32.95 | 10.30 | 14.08 | 14.09 | 0.072 | 0.072 |
| **rno-miR-92a** | 20.86 | 21.96 | 21.96 | 0.16 | 3.07 | 3.10 | 0.133 | 0.130 |
| **rno-miR-224** | 32.89 | 33.96 | 33.97 | 12.19 | 15.06 | 15.11 | 0.137 | 0.132 |
| **rno-miR-145** | 23.91 | 25.00 | 24.97 | 3.21 | 6.10 | 6.11 | 0.135 | 0.134 |
| **rno-miR-328a** | 24.94 | 23.99 | 26.00 | 4.24 | 5.09 | 7.14 | 0.555 | 0.134 |
| **rno-miR-351** | 27.93 | 28.97 | 28.98 | 7.23 | 10.08 | 10.12 | 0.138 | 0.134 |
| **rno-miR-206** | 21.88 | 24.93 | 22.94 | 1.18 | 6.04 | 4.08 | 0.035 | 0.135 |
| **rno-miR-191** | 16.95 | 17.97 | 17.99 | -3.75 | -0.93 | -0.87 | 0.141 | 0.136 |
| **rno-miR-322*** | 26.93 | 27.95 | 27.95 | 6.23 | 9.06 | 9.10 | 0.141 | 0.137 |
| **rno-miR-338*** | 31.98 | 32.99 | 32.99 | 11.28 | 14.10 | 14.14 | 0.141 | 0.138 |
| **rno-miR-434** | 25.99 | 26.98 | 26.99 | 5.29 | 8.09 | 8.13 | 0.144 | 0.139 |
| **rno-miR-99b** | 29.98 | 31.90 | 30.98 | 9.28 | 13.01 | 12.12 | 0.076 | 0.140 |
| **rno-miR-133b** | 20.99 | 23.99 | 21.99 | 0.29 | 5.10 | 3.13 | 0.036 | 0.140 |
| **rno-miR-133a** | 16.99 | 18.96 | 17.98 | -3.71 | 0.07 | -0.88 | 0.073 | 0.141 |
| **rno-miR-205** | 29.02 | 29.82 | 30.01 | 8.32 | 10.93 | 11.15 | 0.165 | 0.141 |
| **rno-miR-34b*** | 28.99 | 30.01 | 29.95 | 8.29 | 11.11 | 11.09 | 0.141 | 0.143 |
| **rno-miR-223** | 17.97 | 19.96 | 18.92 | -2.73 | 1.07 | 0.06 | 0.072 | 0.145 |
| **rno-miR-219-2-3p** | 23.78 | Undet | Undet |  |  |  |  |  |
| **rno-miR-208*** | 24.65 | Undet | Undet |  |  |  |  |  |
| **rno-miR-29b-1*** | 24.90 | Undet | Undet |  |  |  |  |  |
| **rno-miR-219-5p** | 25.58 | Undet | Undet |  |  |  |  |  |
| **rno-miR-186*** | 25.86 | Undet | Undet |  |  |  |  |  |
| **rno-miR-298** | 25.86 | Undet | Undet |  |  |  |  |  |
| **rno-miR-211** | 26.71 | Undet | Undet |  |  |  |  |  |
| **rno-miR-673*** | 26.79 | Undet | Undet |  |  |  |  |  |
| **rno-miR-409-5p** | 26.96 | Undet | Undet |  |  |  |  |  |
| **rno-miR-216a** | 27.82 | Undet | Undet |  |  |  |  |  |
| **rno-miR-876** | 27.90 | Undet | Undet |  |  |  |  |  |
| **rno-let-7b*** | 28.87 | Undet | Undet |  |  |  |  |  |
| **rno-miR-329** | 29.81 | Undet | Undet |  |  |  |  |  |
| rno-miR-139-5p | 27.93 | 27.97 | 29.97 | 7.23 | 9.07 | 11.11 | 0.279 | 0.068 |
| rno-miR-134 | 27.91 | 28.04 | 28.99 | 7.22 | 9.14 | 10.14 | 0.263 | 0.132 |
| rno-miR-675* | 28.97 | 29.00 | 30.00 | 8.27 | 10.11 | 11.14 | 0.279 | 0.136 |
| rno-miR-382 | 30.03 | 29.02 | 30.96 | 9.33 | 10.12 | 12.10 | 0.575 | 0.146 |
| rno-miR-423 | 24.86 | 25.94 | 24.94 | 4.16 | 7.05 | 6.08 | 0.135 | 0.265 |
| rno-miR-130b | 28.96 | 29.99 | 29.03 | 8.26 | 11.09 | 10.17 | 0.141 | 0.266 |
| rno-miR-340-3p | 32.93 | 33.95 | 32.97 | 12.23 | 15.06 | 14.11 | 0.141 | 0.272 |
| rno-miR-142-5p | 29.99 | 32.00 | 30.00 | 9.29 | 13.10 | 11.14 | 0.071 | 0.277 |
| rno-miR-125a-5p | 29.95 | 31.98 | 29.96 | 9.25 | 13.09 | 11.10 | 0.070 | 0.277 |
| rno-miR-450a | 30.01 | 31.01 | 30.02 | 9.31 | 12.12 | 11.16 | 0.143 | 0.277 |
| rno-miR-127 | 29.98 | 30.98 | 29.98 | 9.28 | 12.08 | 11.12 | 0.143 | 0.279 |
| rno-miR-664 | 27.99 | 28.96 | 27.95 | 7.29 | 10.07 | 9.10 | 0.146 | 0.286 |
| rno-miR-150 | 21.02 | 21.98 | 20.97 | 0.32 | 3.08 | 2.11 | 0.148 | 0.290 |
| rno-miR-296* | 31.00 | 32.05 | 30.95 | 10.30 | 13.16 | 12.09 | 0.138 | 0.290 |
| rno-miR-218 | 29.93 | 30.94 | 28.98 | 9.23 | 12.05 | 10.12 | 0.142 | 0.539 |
| rno-miR-7a | 34.93 | 35.87 | 33.95 | 14.23 | 16.98 | 15.10 | 0.149 | 0.548 |
| rno-miR-1 | 21.97 | 23.98 | 20.98 | 1.27 | 5.08 | 2.12 | 0.071 | 0.554 |
| rno-miR-100 | 29.94 | 30.95 | 28.92 | 9.24 | 12.06 | 10.06 | 0.142 | 0.564 |
| rno-miR-361 | 28.97 | 30.01 | 27.96 | 8.28 | 11.12 | 9.10 | 0.140 | 0.565 |
| rno-miR-190 | 28.87 | Undet | 35.99 |  |  |  |  |  |

The miRNA species that dysregulated commonly in 2 DILI groups are listed in bold characters, and the miRNA species that dysregulated in single DILI group are listed in normal characters. “Undet” is short for “Undetectable”.
