# Supplementary material for: A Panel of Serum MicroRNAs as Specific Biomarkers for Diagnosis of Compound- and Herb-Induced Liver Injury in Rats
Source: PLoS One. 2012 May 18;7(5):e37395. doi: 10.1371/journal.pone.0037395 (PMC3356255; doi:10.1371/journal.pone.0037395)
Supplement: Table S3 — Dysregulated liver tissue miRNAs in 2 DILI groups compared with vehicle group. (DOC) [file pone.0037395.s006.doc]

**Supplementary Data Table 3.** Dysregulated liver tissue miRNAs in 2 DILI groups compared with vehicle group (Fold Change > 1.30 or < 0.77, *P*-value < 0.05)

|  | **APAP vs Vehicle** | | **DB vs Vehicle** | |
| --- | --- | --- | --- | --- |
| **up-regulated miRNAs** | **Fold change** | ***P*-Value** | **Fold change** | ***P*-Value** |
| rno-miR-20a | 1.69 | 0.01195 | 2.17 | 0.00040 |
| rno-miR-17-5p | 1.52 | 0.01586 | 2.07 | 0.00086 |
| rno-miR-7a | 1.43 | 0.00263 | 2.60 | 0.00002 |
| rno-miR-191 | 1.31 | 0.04551 | 1.48 | 0.00328 |
| **down-regulated miRNAs** | **Fold change** | ***P*-Value** | **Fold change** | ***P*-Value** |
| rno-miR-365 | 0.65 | 0.00069 | 0.60 | 0.01433 |
| rno-miR-24-1* | 0.57 | 0.00912 | 0.65 | 0.01578 |
| rno-miR-664 | 0.57 | 0.00307 | 0.71 | 0.00201 |
| rno-miR-99a | 0.52 | 0.02037 | 0.56 | 0.04054 |
| rno-miR-199a-3p | 0.50 | 0.03935 | 0.45 | 0.04238 |
| rno-let-7d* | 0.45 | 0.02824 | 0.47 | 0.04093 |
| rno-miR-214 | 0.32 | 0.03051 | 0.37 | 0.02866 |
| rno-miR-363* | 0.22 | 0.00205 | 0.38 | 0.03999 |

The raw data of microarray hybridization is MIAME compliant and has been deposited in ArrayExpress, Gene Expression Omnibus (Accession Number: E-MEXP-3364).
