# Supplementary material for: A Panel of Serum MicroRNAs as Specific Biomarkers for Diagnosis of Compound- and Herb-Induced Liver Injury in Rats
Source: PLoS One. 2012 May 18;7(5):e37395. doi: 10.1371/journal.pone.0037395 (PMC3356255; doi:10.1371/journal.pone.0037395)
Supplement: Table S4 — qRT-PCR validation of dysregulated liver miRNAs in microarray hybridization results. (DOC) [file pone.0037395.s007.doc]

**Supplementary Data Table 4.** qRT-PCR validation of dysregulated liver miRNAs in microarray hybridization results.

| **miRNA** | **APAP vs Vehicle** | | **DB vs Vehicle** | | **Consistent with liver tissue**  **hybridization results** | **Consistent with serum**  **TLDA results** |
| --- | --- | --- | --- | --- | --- | --- |
|  | **Fold change** | ***P*-value** | **Fold change** | ***P*-value** |  |  |
| rno-miR-7a | 1.75 | 0.02240 | 1.38 | 0.42991 | Yes | No |
| rno-miR-20a | 1.34 | 0.00074 | 1.37 | 0.00911 | Yes | Yes |
| rno-miR-17-5p | 1.26 | 0.30094 | 1.08 | 0.68274 | Yes | No |
| rno-miR-191 | 0.94 | 0.60727 | 0.71 | 0.08754 | No | Yes |
| rno-miR-99a | 0.90 | 0.26931 | 0.49 | 0.00940 | Yes | Yes |
| rno-miR-664 | 0.85 | 0.28238 | 0.53 | 0.08541 | Yes | Yes |
| rno-miR-365 | 0.83 | 0.19726 | 0.52 | 0.00954 | Yes | No |
| rno-miR-199a-3p | 0.82 | 0.15823 | 0.55 | 0.06343 | Yes | Yes |
| rno-miR-363* | 0.79 | 0.17662 | 0.92 | 0.90930 | Yes | - |
| rno-miR-214 | 0.72 | 0.26717 | 0.35 | 0.06920 | Yes | Yes |
| rno-miR-24-1* | 0.72 | 0.16228 | 0.42 | 0.00904 | Yes | - |
| rno-let-7d* | 0.49 | 0.44445 | 0.25 | 0.11216 | Yes | - |
| rno-mir-122 ^#^ | 0.82 | 0.18743 | 0.48 | 0.01031 | Yes | No |
| rno-mir-192 ^#^ | 0.74 | 0.44445 | 0.46 | 0.02613 | Yes | No |
| rno-mir-193 ^#^ | 0.86 | 0.55841 | 0.60 | 0.06792 | Yes | No |

# The expression levels of liver tissue miRNA-122, miRNA-192 and miRNA-193 had also been validated.
