# Supplementary material for: A Panel of Serum MicroRNAs as Specific Biomarkers for Diagnosis of Compound- and Herb-Induced Liver Injury in Rats
Source: PLoS One. 2012 May 18;7(5):e37395. doi: 10.1371/journal.pone.0037395 (PMC3356255; doi:10.1371/journal.pone.0037395)
Supplement: Table S5 — Sequences of synthetic mature miRNAs. (DOC) [file pone.0037395.s008.doc]

**Supplementary Data Table 5.** Sequences of synthetic mature miRNAs.

| **Mature miRNA accession** | **Mature miRNA ID** | **Synthetic mature miRNA sequence** |
| --- | --- | --- |
| MIMAT0000827 | rno-miR-122 | UGGAGUGUGACAAUGGUGUUUG |
| MIMAT0000867 | rno-miR-192 | CUGACCUAUGAAUUGACAGCC |
| MIMAT0000868 | rno-miR-193 | AACUGGCCUACAAAGUCCCAGU |
