# Supplementary material for: A Panel of Serum MicroRNAs as Specific Biomarkers for Diagnosis of Compound- and Herb-Induced Liver Injury in Rats
Source: PLoS One. 2012 May 18;7(5):e37395. doi: 10.1371/journal.pone.0037395 (PMC3356255; doi:10.1371/journal.pone.0037395)
Supplement: Table S6 — Top 20 abundant miRNA species of liver tissue in microarray hybridization results. (DOC) [file pone.0037395.s009.doc]

**Supplementary Data Table 6.** Top 20 abundant miRNA species of liver tissue in microarray hybridization results.

| **Rank** | **miRNA** | **Hybridization intensity (vehicle group, n=3)** | |
| --- | --- | --- | --- |
|  |  | **Mean** | **SD** |
| 1 | rno-miR-22 | 37501.33 | 3442.01 |
| 2 | rno-miR-21 | 26249.33 | 1619.10 |
| **3** | **rno-miR-122** | **19575.50** | **691.79** |
| 4 | rno-miR-26a | 16379.50 | 3903.30 |
| 5 | rno-miR-126 | 10125.00 | 1645.14 |
| 6 | rno-miR-29a | 10064.67 | 2026.70 |
| **7** | **rno-miR-193** | **9557.67** | **913.11** |
| 8 | rno-miR-101b | 7711.67 | 252.79 |
| 9 | rno-miR-378 | 7355.67 | 377.74 |
| 10 | rno-miR-101a | 7302.67 | 455.52 |
| 11 | rno-miR-30b-5p | 7093.83 | 1079.78 |
| 12 | rno-miR-23b | 5836.50 | 959.42 |
| 13 | rno-miR-451 | 5175.00 | 655.38 |
| 14 | rno-let-7a | 4722.67 | 798.36 |
| 15 | rno-miR-16 | 4684.67 | 360.87 |
| 16 | rno-miR-142-3p | 4671.33 | 1298.65 |
| 17 | rno-miR-194 | 4653.83 | 773.43 |
| **18** | **rno-miR-192** | **4512.50** | **417.03** |
| 19 | rno-let-7b | 3903.83 | 742.46 |
| 20 | rno-miR-30c | 3706.17 | 322.88 |

The raw data of microarray hybridization is MIAME compliant and has been deposited in ArrayExpress, Gene Expression Omnibus (Accession Number: E-MEXP-3364).
